# Post-COVID-19 Benign Paroxysmal Positional Vertigo

**DOI:** 10.1155/2021/9967555

**Published:** 2021-06-01

**Authors:** Siniša Maslovara, Andro Košec

**Affiliations:** ^1^Department of Otorhinolaryngology and Neurosurgery, Faculty of Dental Medicine and Health, University of Osijek, Osijek, Croatia; ^2^Department of Otorhinolaryngology, National Memorial Hospital, Vukovar, Croatia; ^3^Department of Otorhinolaryngology and Head and Neck Surgery, Zagreb School of Medicine, University Hospital Center “Sestre Milosrdnice”, Zagreb, Croatia

## Abstract

**Background:**

COVID-19 is a disease caused by a new strain of coronavirus (SARS-COVID-19). The WHO first learned about this new virus on December 31, 2019, following a report on a group of cases of “viral pneumonia” in Wuhan, People's Republic of China, and declared a pandemic in March 2020. The disease was an almost complete unknown at the outset, but knowledge of its pathophysiology, clinical picture, and treatment options grew and developed rapidly.

**Objective:**

Dizziness is a very common symptom that patients report in routine clinical practice and one of the significant clinical manifestations of COVID-19. This short report investigated a possible connection between COVID-19 and benign paroxysmal positional vertigo (BPPV).

**Methods:**

We present two cases of benign paroxysmal positional vertigo (BPPV), which developed within two weeks of SARS-COVID-19 infection, manifesting with mild disease severity in early November 2020.

**Results:**

In both cases, the disease manifested by severe, short-lived attacks of vertigo, objective-circular type, with accompanying nausea and vomiting. The symptoms occurred when lying in bed and turning to the right and assuming an upright position. The diagnosis of posterior semicircular canal BPPV (PC-BPPV) was made based on a subjectively and objectively positive right-sided Dix–Hallpike test.

**Conclusions:**

The pathophysiology of post-COVID BPPV is probably similar to that of other viral infections, with some of its specificities such as inducing hypercoagulation and microthrombus formation, which can cause significant circulatory disorders possibly affecting its pathogenesis.

## 1. Introduction

Coronavirus disease-19 (COVID-19) is caused by severe acute respiratory syndrome coronavirus 2 (SARS-COVID-19). The WHO declared a pandemic of this virus in March 2020 after its rapid spread from the city of Wuhan, in the province of Hubei in China [1]. It has been shown that, in addition to the most common clinical symptoms of the disease, such as dry cough, fever, and general weakness, some patients develop taste and smell disorders, headaches, congestion of the nasal mucosa, gastrointestinal disorders, and dizziness. A small proportion of patients may develop significant complications such as bilateral pneumonia, accompanied by shortness of breath, chest pain, and a characteristic finding of ground-glass opacity on CT, which indicates severe viral pneumonia [2, 3].

The infection can affect the central nervous system, and neurological symptoms can appear both in the initial disease stages and in the protracted recovery period. Most patients experience, to a greater or lesser extent, neurological and psychological symptoms, ranging from insomnia, anxiety, and depression to disturbances of consciousness, confusion, and epileptic seizures.

Among neurological complications, the most common are encephalopathy and cerebrovascular stroke, disorders of consciousness, seizures, and hypoxic brain injuries. Dysosmia and dysgeusia and nonspecific symptoms such as headache, dizziness, and paresthesia have been reported. Dizziness is a very common symptom that patients report in routine clinical practice [4].

Numerous studies, appearing daily from various parts of the world, have revealed vertigo as one of the significant clinical manifestations of COVID-19. One of them cites vertigo as the most common neurological manifestation of COVID-19, thought to follow the neuroinvasive potential of the virus [5]. Some researchers hypothesized that the virus enters neuronal tissue from the circulation and binds angiotensin-converting enzyme 2 receptors (ACE2), which are located in the capillary endothelium. Other mechanisms that lead to dizziness during COVID-19 infection are direct inflammatory action of the virus on nervous tissue, indirect immune response, hypoxia, and hypercoagulopathy [5].

After performing a literature search of *PubMed* and *Google Scholar* databases to identify published cases of vertigo associated with COVID-19, three case reports and 11 studies listing vertigo and COVID-19 were identified. Out of these 14 studies, only two investigated vertigo as a presenting symptom, and only one noted vestibular rehabilitation treatment details and disease outcome [6]. Vertigo presented as an initial COVID-19 symptom in three patients and followed respiratory symptoms in two patients [7].

The most important element in this case report is the consideration of possible causes and disease course of post-COVID BPPV. A thorough literature review shows that BPPV may be associated with COVID-19 by a specific mechanism of etiopathogenesis.

Our case report was assembled following CARE (CAse REport) guidelines and the Institutional Review Board according to the Helsinki Declaration of 1983. Written informed consent for publication was obtained from both patients.

## 2. Case Presentation

### 2.1. Case 1

This report lists two very similar cases of post-COVID-19 BPPV. The first is a 41-year-old female patient that was diagnosed with COVID-19 in early November 2020. She was tested for SARS-COVID-19 using a PCR test (nasopharyngeal swab) positive on November 5 and 20, 2020, and tested negative on November 30, 2020. Apart from fever up to 37.2 °C and severe pain in her legs, she did not develop most of the common symptoms of COVID-19, such as high fever, dry cough, shortness of breath, and a sudden loss of smell and taste. She did not receive any drugs, and a chest CT scan was not performed because she did not have any significant respiratory problems. She returned to work in early December, and a week later developed severe, short-lived attacks of objectively circular dizziness, usually when getting out of bed or turning on her right side that lasted for six days, followed by nausea and vomiting. Initially, she also had severe occipital headaches that spread to her neck, which subsided after the first few days of illness. Upon initial examination by her GP, she was referred to a neurologist. Dizziness subsided after the first few days of illness. Among the laboratory findings, only serum K+ level was decreased (3.7 mmol/L), attributed to a loss of body fluid by vomiting. She was afebrile, normotensive, independently mobile, and eupneic. Her neurological status was normal. The neurologist concluded that it is a case of peripheral vertigo and referred the patient to an otorhinolaryngologist. The patient did not have hearing problems, denied tinnitus, and complained of a moderate occipital headache. She was on bisoprolol therapy previously due to tachycardia, diagnosed after her third pregnancy in 2017. A neurotological assessment showed negative spontaneous nystagmus, and head impulse and skew deviation tests were also negative. The Romberg test with the open and closed eyes was without lateralization, as well as the Fukuda test. A diagnosis of right-sided posterior semicircular canal canalithiasis (PC-BPPV) was made based on a positive Dix–Hallpike test [8, 9]. In the right position, after a latency of ten seconds, dizziness and nausea occurred, and objectively, a small vertical-torsional geotropically directed positional nystagmus (PN) was observed, lasting for 5 seconds. When returning to a sitting position, dizziness increased, with nausea and PN becoming more pronounced. A right-sided Epley repositioning procedure was performed twice in one session, after which the patient no longer had symptoms [10]. The patient was alerted to the possible occurrence of an otolithic crisis during the first 24 h after performing the Epley procedure. The patient was advised to test her serum vitamin D3 levels and to follow up in a week.

### 2.2. Case 2

Similarly, the second 28-year-old female patient was also diagnosed with a mild form of COVID-19 on November 7 and tested negative on November 20, 2020. She underwent an antigen test using the SARS-COVID-19 IgG method: Abbott Architect i2000-CMIA 18.12. 2020, which was positive, at the level of 8.18 (recommended values: <1.4). In the first days of the disease, the dominant symptom was a moderate frontal headache, followed by muscle and joint pain, diarrhea, vomiting, and general weakness. Therefore, she occasionally took 400 mg of ibuprofen p.o. As with the former patient, she did not experience the most common symptoms of coronavirus disease, such as fever, dry cough, shortness of breath, loss of smell and taste, or a change in taste. Short-lived, but intense attacks of objectively circular dizziness, accompanied by nausea when turning to the right side and getting out of bed, occurred on December 20, 2020, about one month after being diagnosed with COVID-19. She is otherwise healthy, does not have hearing complaints, denies tinnitus, and sometimes has moderate frontoparietal headaches. During the clinical examination, a test of spontaneous nystagmus, head impulse, and skew deviation were negative. The Romberg test with the open and closed eyes was without lateralization, as well as the Fukuda test. The Dix–Hallpike test was subjectively and objectively positive on the right side upon a return to the sitting position. After a latency of a few seconds, dizziness and nausea occurred, and a rough, geotropically oriented vertical-torsional PN is observed, lasting about 15 seconds. A diagnosis of PC-BPPV was made, and a right-sided Epley repositioning procedure was performed on two occasions in one session. After the second iteration, the patient was symptom-free, and PN could not be observed. This patient was also advised to test her serum vitamin D3 levels and to follow up in a week. The patient was also alerted to the possible occurrence of an otolithic crisis during the first 24 h after performing the Epley procedure.

## 3. Discussion

The COVID-19 pandemic has brought with it many new issues and intensified many previously known health-related, economic, and social problems. The disease is predominantly characterized by an acute respiratory disorder but also causes several neurological symptoms, including disorders of smell and taste, headache, and dizziness (up to 20% of patients). Treatment of the infection itself has so far been largely limited to attempts to coadminister azithromycin and hydroxychloroquine, as well as symptomatic treatment and management of complications, while corticosteroids are used often, but mostly nonrationally, as they should be reserved for more severe disease instances [11]. Similarly, treatment of vertigo during COVID-19 is primarily concerned with symptomatic treatment using antiemetics, antihistamines, and sedatives.

The emergence of post-COVID-19 vertigo, alongside other post-COVID-19 conditions, is a new development. In this article, we presented two very similar cases of PC-BPPV a few weeks after overcoming the milder clinical form of COVID-19. In the future, we can expect a greater number of post-COVID vertigo patients, and the differential diagnosis of acute-onset vertigo should include BPPV and vestibular neuronitis as a consequence of SARS-COVID-19 infection. In the case of chronic persistent postural-perceptive vertigo (PPPD) and other functional vestibular disorders, a significant increase in patients seen by vestibular specialists may also be expected, due to the pronounced impact of the COVID-19 pandemic on both physical and mental health.

BPPV represents 17.1% of the total number of dizziness complaints in the general population, while in the elderly this share rises to approximately 50% [12, 13]. von Brevern et al. found that BPPV is responsible for 8% of moderate or severe vertigo, with a lifetime prevalence of 2.4%, while a one-year prevalence is 1.6%, and an incidence of 0.6% [14].

The BPPV pathophysiology may be explained by two cardinal theories, cupulolithiasis and canalithiasis. The understanding of metabolic mechanisms that regulate the level of calcium, proteins, and mucopolysaccharides involved in the construction of the otolithic membrane, which disrupts the rupture of adhesive bonds that hold otoliths together may also shed new light on the emergence of post-COVID BPPV [15–17]. It is known that the elderly suffer the most from BPPV due to degenerative changes in the macula of the utriculus, probably due to lower calcium levels in the endolymph and reduced number and volume of otoliths. Most cases of BPPV are idiopathic in origin and probably result from a degeneration of the macula. The secondary BPPV causes refer to the identifiable causes of otoconial dislodgement. Apart from otologic and nonotologic surgery, head trauma, vestibular neuritis, Meniere's disease, and a sudden sensorineural hearing loss may also cause the BPPV. However, in younger patients, such as our two cases, other pathophysiological mechanisms come into consideration [18, 19].

Viral labyrinthitis is responsible for the occurrence of about 15% of BPPV which occurs due to the detachment of otoliths from the utriculus, either by the direct action of inflammatory elements near the macula or indirectly, due to disorders of labyrinthine microcirculation, representing viral ganglionitis. Neuropathologic changes (focal axonal degeneration and vestibular ganglion cell loss) and a high incidence of cupular basophilic deposits seen in the patients' temporal bones with a known BPPV weaken a purely mechanistic concept of BPPV symptoms. A recently reported quantitative study involving five patients with the BPPV has revealed a significant degeneration (50%) of both the superior and the inferior divisions of the vestibular ganglion and a small cupular deposit in only one patient, supporting neural injury as the principal etiopathogenetic pathway, easily caused by viral neurotoxicity [19, 20]. In the case of COVID-19, it is a very specific viral infection, accompanied by the formation of microthrombi in the circulation, possibly explaining the occurrence of BPPV after the initial phase of the SARS-COVID-19 infection. However, as the blood supply to the inner ear courses through the *a. labyrinthi* (*a. auditoria interna*), the end branch of the anterior inferior cerebellar artery (*a. cerebelli anterior inferior*-AICA), its loss of function would cause hearing loss, which challenges this concept of BPPV attributed to microcirculation issues. However, there are reports of otoconia being displaced due to vasospasms in the circulation of the inner ear [21]. The mechanisms behind the otoconia degeneration and displacement are still unclear. The proinflammatory regulators are activated during a viral inflammatory process. Nuclear factor kappa-light-chain-enhancer of the activated B cells (NF-*κ*B) is one of the most widely known mediators controlling proinflammatory signaling and has been correlated with an increased expression in various inflammatory conditions, including viral infections such as the COVID-19 [22]. A possible cause of post-COVID BPPV could be prolonged bed rest, as it has long been known that otoconia rupture can occur due to insufficient movement, probably due to hypercoagulability. Although Mao et al. state that neurological symptoms occur much more frequently in patients with a more severe acute respiratory syndrome, both of our patients had a very mild COVID-19 and yet developed BPPV a few weeks after the resolution of disease symptoms [23].

As prevention of recurrence of BPPV, and with limited rationale, in preventing severe forms of COVID-19, vitamin D3 is used to a greater extent worldwide, due to its low level in the general population, and also very small side effects [24, 25].

Although the neurotropic nature of SARS-COVID-19 has been known since the beginning of the pandemic, data on neurologic symptoms have been scarce to date, save dysosmia. Few studies evaluated vertigo as a presenting symptom. There is currently no evidence of post-COVID-19 BPPV in the available medical literature, and this report aims to encourage others to evaluate post-COVID-19 patients presenting with vestibular symptoms comprehensively, reducing the number of delayed referrals and misdiagnosed patients.

## Data Availability

The data used to support the findings of this study are available from the corresponding author upon request.
